# PtRFdb: a database for plant transfer RNA-derived fragments

**DOI:** 10.1093/database/bay063

**Published:** 2018-06-22

**Authors:** Nikita Gupta, Ajeet Singh, Shafaque Zahra, Shailesh Kumar

**Affiliations:** Lab #202, National Institute of Plant Genome Research (NIPGR), Aruna Asaf Ali Marg, New Delhi, India

## Abstract

Transfer RNA-derived fragments (tRFs) represent a novel class of small RNAs (sRNAs) generated through endonucleolytic cleavage of both mature and precursor transfer RNAs (tRNAs). These 14–28 nt length tRFs that have been extensively studied in animal kingdom are to be explored in plants. In this study, we introduce a database of plant tRFs named PtRFdb (www.nipgr.res.in/PtRFdb), for the scientific community. We analyzed a total of 1344 sRNA sequencing datasets of 10 different plant species and identified a total of 5607 unique tRFs (758 tRF-1, 2269 tRF-3 and 2580 tRF-5), represented by 487 765 entries. In PtRFdb, detailed and comprehensive information is available for each tRF entry. Apart from the core information consisting of the tRF type, anticodon, source organism, tissue, sequence and the genomic location; additional information like PubMed identifier (PMID), Sample accession number (GSM), sequence length and frequency relevant to the tRFs may be of high utility to the user. Two different types of search modules (Basic Search and Advanced Search), sequence similarity search (by BLAST) and Browse option with data download facility for each search is provided in this database. We believe that PtRFdb is a unique database of its kind and it will be beneficial in the validation and further characterization of plant tRFs.

Database URL: http://www.nipgr.res.in/PtRFdb/

## Introduction

**Figure 1.**
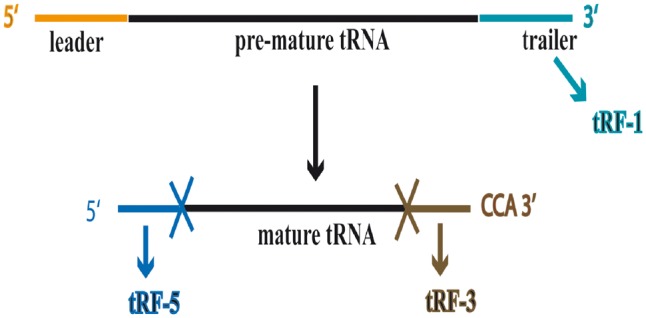
Biogenesis of different types of tRFs from precursor and mature tRNAs.

Advancements in high throughput sequencing technologies led to the identification of various types of small RNAs (sRNAs) involved in the regulation of diverse biological processes (1, 2). Functional roles of these regulatory sRNAs have been well described in plant genomics (3). Micro-RNAs are the most abundant and the most extensively studied sRNAs, which are the key molecules governing development and stress responses in plants (4, 5). With advancement of techniques, sRNAs originating from non-coding RNAs (ncRNAs) such as ribosomal RNAs (rRNAs), small nuclear RNAs (snRNAs), small nucleolar RNAs (snoRNAs) and transfer RNAs (tRNAs) have also been discovered. Such sRNAs are not random degradation products as these have specific processing and post-processing functions (6–8). Currently, sRNAs originating from tRNAs that are analogous to miRNAs are gaining particular attention (9). There are two major classes of sRNAs originating from tRNAs i.e. 30–36nt long tRNA halves and 14–26 nt long tRNA-derived fragments (tRFs) (10–16).tRFs constitute a distinct group of sRNAs generated by endonucleolytic cleavage of both mature and precursor tRNAs. tRFs being extensively studied in the animal kingdom have recently been realized to be of significance in plant genomics (10, 13). Current understanding of the plant tRFs indicates that these sRNAs originate from 5′ end (i.e. tRF-5) and 3′ end (i.e. tRF-3) of mature tRNAs; and precursor tRNAs (i.e. tRF-1) (Figure 1) with variable length in the range 15–28 nt (15). tRFs derived from mature tRNAs are more in number as compared to the tRFs produced from tRNA precursors (i.e. pre-tRNAs) (12). In plants, there is an enhancement of tRFs generation in case of different biotic (14, 17) and abiotic (15, 18, 19) stress conditions. *In silico* and *in vivo* expression analyses performed by Alves *et al.* (13) indicate that tRFs are merely not a by-product of tRNA degradation and their abundance does not correlate with the number of genomic copies of the parent tRNAs. The same study also showed that the tRF-5 is the most abundant type of tRFs.

Although both Dicer-dependent and Dicer-independent pathways have been studied for tRFs production in animals (20), in plants it has been shown that tRFs are associated with Dicer-like 1 (DCL1) enzymes (10) which are indispensable for the micro-RNAs generation, thus suggesting that the biogenesis of tRFs follows the canonical pathway of micro-RNA processing. Studies performed using the model plant *Arabidopsis thaliana* have confirmed that certain tRFs are associated with Argonaute proteins (AGO 1, 2, 4, 7) and using *in silico* approaches their putative target genes have also been predicted (21). This reflects that tRFs have a clear role to play in post-transcriptional gene silencing (PTGS) showing functional similarities with micro-RNAs. However, the predicted target genes of specific tRFs need experimental validation to confirm their role in gene regulation in plants. Moreover, the tRFs have been found to be involved in the translation repression (22) and genome stability by controlling post-transcriptional process of retrotransposons (23). Recent reports suggest the role of tRFs in root nodule and arbuscular mycorrhiza formation in leguminous plants (24).

**Figure 2.**
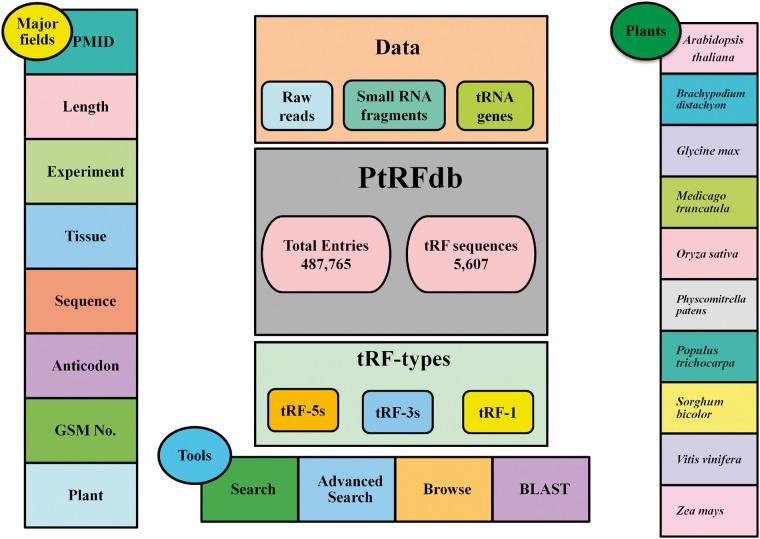
Overall representation of PtRFdb database.

The availability of a knowledgebase dedicated to the plant tRFs is anticipated to assist the exploration of tRFs-related information, biogenesis and functions in various plants. Currently available tRFs databases like tRFdb (25), tRF2Cancer (26) and MINTbase (27) harbor information about tRFs present in various organisms. With best of the knowledge of the authors, a freely available plant tRFs database is currently unavailable. In this study, we introduce a database of plant tRFs named PtRFdb (http://www.nipgr.ac.in/PtRFdb/), for plant research community. The overall representation of the database is illustrated in Figure 2. We have also collected the information of experimentally identified tRFs available in literature from *A. thaliana*, *Medicago truncatula*, *Oryza sativa*, *Piper nigum* and *Triticum aestivum*. The information has been provided as a downloadable excel sheet (Supplementary File S1).

## Materials and methods

### Database preparation

We downloaded the tRNA genes of the species *A. thaliana* (TAIR10 February 2011), *Brachypodium distachyon* (JGI v1.0 8X), *Glycine max* (Wm82.a2), *M. truncatula* (March 2009 Version 3.0), *O. sativa* (v7.0), *Physcomitrella patens* (Version 1.1), *Populus trichocarpa* (January 2010 Version 2.0), *Sorghum bicolor* (Version 1.0), *Vitis vinifera* (Grapevine 12X) and *Zea mays* (Version 5b.60) from GtRNAdb (28) and their reference genomes from the corresponding genomic portals (e.g. *A. thaliana* genome from https://www.araport.org). For each plant species, FASTA sequences of tRNA genes were extracted according to the strand information of tRNA gene transcription. Since tRNA nucleotidyltransferases (CCA-adding enzymes) are responsible for maturation of the functional 3' end of tRNA, therefore ‘CCA’ was manually added to tRNA sequences procured from tRNAscan-SE to obtain mature tRNA sequences. To identify the tRFs generated from pre-tRNAs, we have also extracted the sequences that spanned 40 nt upstream and 40 nt downstream to the mature tRNA genes. Finally, a reference database of each plant species was made by combining both mature and pre-tRNA sequences (tRNA gene with 40 nt upstream and 40 nt downstream) using ‘makeblastdb’ script of Basic Local Alignment Search Tool (BLAST) v 2.6.0 (29). This database was further used for the identification of three types of tRFs (i.e. tRF-5, tRF-3 and tRF-1) in each plant species.

### Datasets

For each plant, we have searched Gene Expression Omnibus (GEO) repository of NCBI (http://www.ncbi.nlm.nih.gov/geo/) by using the search terms ‘small RNA sequencing’ and ‘miRNA sequencing’ and downloaded the data of unique sRNA fragments with their clonal frequencies in different experiments. In case of non-availability of the unique RNA sequences, raw sequencing reads of those experiments were downloaded from Sequence Read Archive (SRA) of NCBI (https://www.ncbi.nlm.nih.gov/sra). Conclusively, we have processed two types of datasets, i.e. small/miRNA data of unique sequences with clonal frequencies and datasets in form of raw sequencing reads (i.e. FASTQ format).

### Identification of tRFs

**Figure 3.**
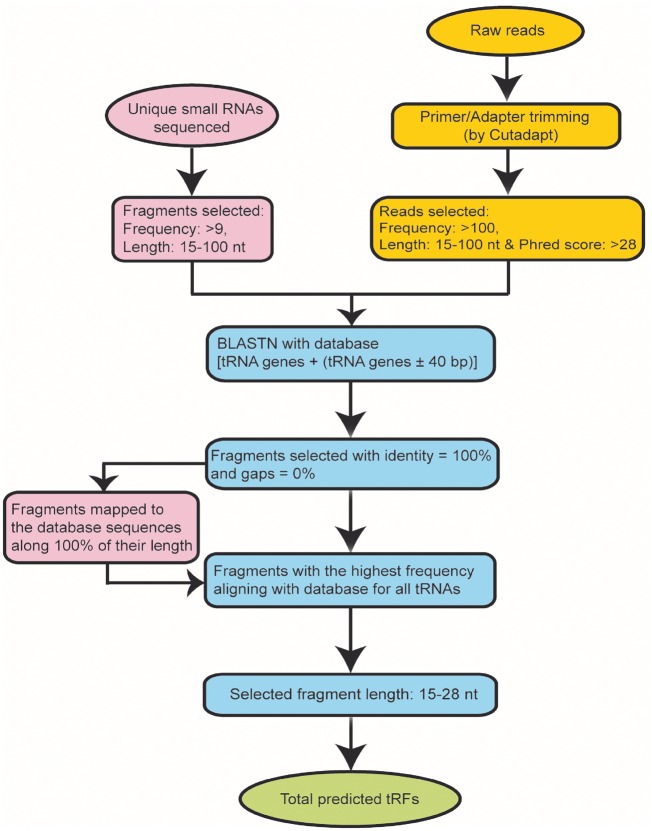
Methodology used for the identification of tRFs.

For unique sRNA fragment data, we selected the fragments of length 15 28 nt having the clonal frequency >9 and aligned them to the tRNA database by using BLASTN. Unique fragments mapped along 100% of their length were only considered for further analysis (Figure 3).

Raw sequencing data generated by Illumina sequencing technology was processed for the removal of primer and adaptor sequences with Cutadapt v1.14 tool (https://cutadapt.readthedocs.io/en/stable/) at default parameters. This tool trimmed the reads by considering the input set of primer and adaptor sequences used in Illumina sequencing technology. We further analyzed the trimmed reads with tDRmapper (30) to discard the reads having base of quality score <28 at any position; and to calculate the frequency of each unique read. Finally, we selected the high-quality reads of length 15–100 nt with frequency >100 and aligned them to the autogenous customized tRNA database by using BLASTN (Figure 3).

For both types of datasets, BLAST hits only with 100% identity and 0% gaps were considered. If any query sequence aligns to both pre-tRNA and mature tRNA gene sequences, then we considered only the hits with mature tRNA gene because pre-tRNA itself contains the sequence of mature tRNA gene (except CCA at 3′ end).

Since the database of FASTA sequences was prepared according to the strand information of tRNA gene, therefore we performed the BLASTN for all the query sequences for the positive strand of subjects (i.e. tRNA databases). BLAST output file was parsed using an in-house developed Perl script to get the mapping coordinates. Further, query sequences with the highest frequency aligning with the database for all tRNAs were extracted to abolish random hits (Figure 3).

Finally, we selected the sequences of length 15–28 nt hitting exactly at 3′ end (i.e. tRF-3), 5′ end (i.e. tRF-5) and sequences starting exactly from the first base pair of 3′ trailer end (i.e. tRF-1) and considered them as tRFs (Figure 3). In our Next Generation Sequencing (NGS) data analysis pipeline, during the assignment of tRFs, we have considered the sequences of length in the range 15–28 nt. This criterion of length was adopted on the basis of tRFs length information mentioned in previous studies (10, 13, 15).

### PtRFdb web interface

Information regarding all the GEO samples analyzed in this study was extracted by the ‘SRAdb’ and ‘GEOmetadb’ libraries of Bioconductor package (http://www.bioconductor.org) and combined with each entry of PtRFdb. After the collection and compilation of all the information, the database was built on an Apache Hypertext Transfer Protocol (HTTP) Server with MySQL. MySQL is an object-relational database management system (RDBMS), and it works at the backend. It provides commands to retrieve and store the data in the database. Hypertext Markup Language (HTML), Hypertext Pre-processor (PHP) and JAVA scripts were used to develop the front–end web interface. All common gateway interface and database interfacing scripts were written in the PHP and PERL programming languages. Because Apache, MySQL and PHP technology are platform-independent and open-source software, these were preferred to develop the database.

### PtRFdb features and data retrieval tools

In PtRFdb, detailed and comprehensive information is incorporated for each tRF entry. Apart from the core information including the tRF type, anticodon, source organism, tissue, sequence and the genomic location; additional information like PMID, GSM, sequence length and frequency relevant to the tRFs may be of high utility to the user.

PtRFdb provides two user-friendly options to search tRFs from different types of keywords. First option ‘SEARCH’ facilitates the user to query the tRFs by providing different search terms including the name of the plant source, tRF type, sequence, anticodon etc. To provide the flexibility, two options i.e. ‘containing’ and ‘exact’ have been incorporated for search terms. This option also facilitates the user to select the fields to be displayed. A total of 10 display fields are available for a particular search term. Three display fields namely the ‘GSM number’, ‘PMID’ and Sequence are further linked with their corresponding information.

Second option to search in PtRFdb is that of the ‘ADVANCED SEARCH’ providing the facility to make the user-built complex query using combinations of keywords. The keywords here can be defined to be included together or searched alternatively or excluded. This is possible using the conditional operator ‘=’ and ‘Like’ and also, the logical operators ‘OR and ‘AND’.

The ‘BROWSE’ section of PtRFdb facilitates the user to extract tRFs information by three different approaches (browse by plant name or tRF type or by anticodon type). A query sequence can be aligned to the tRFs sequences of PtRFdb using the ‘BLAST’ module incorporated in this database. This section has the facility to select the plant to which the user wishes to align the input sequences. Different options of ‘E value’ are also available in this section for BLAST search to know the significance of match. The ‘STATISTICS’ section displays the overall enumeration of tRFs in each plant species.

The ‘METHODS’ section elaborates on all the steps of tRFs identification used for this study i.e. raw data download, data processing, tRNA genes database development, analysis of sRNA fragments and FASTQ sequences and assignment of tRF type. From the ‘HELP’ section of PtRFdb, the user can understand the working of this database with the help of self-explanatory figures.

## Results and Discussion

**Figure 4.**
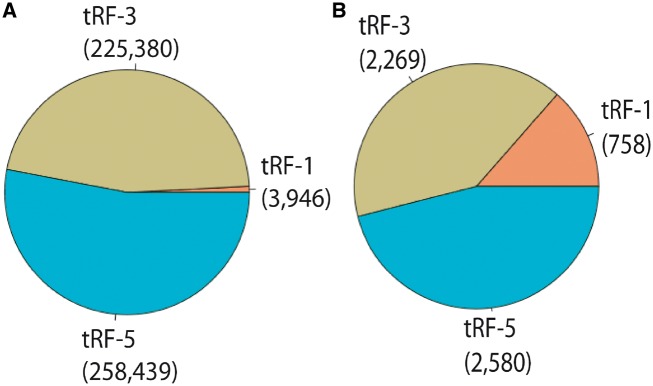
Distribution of different types of tRFs available at PtRFdb, (**A**) total entries of different tRF-types stored in PtRFdb, (**B**) unique tRFs sequences (tRF-5, tRF-3 and tRF-1) in all plant species present in PtRFdb.

**Figure 5.**
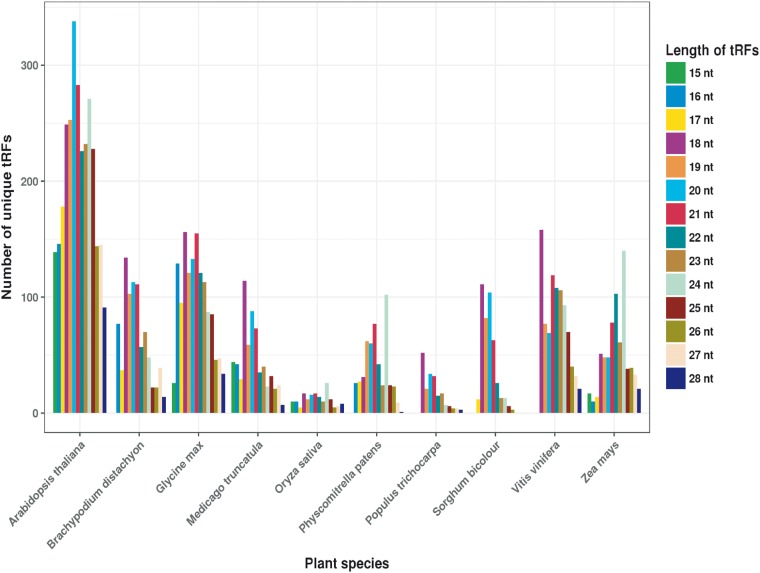
Length-wise distribution of unique tRF sequences identified per plant species.

**Figure 6.**
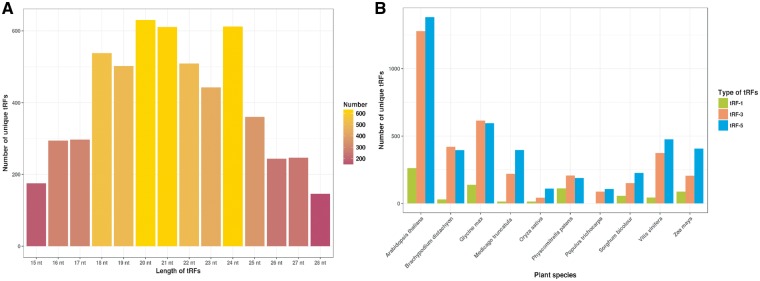
Distribution of unique tRF sequences identified, (**A**) total number of unique tRFs on the basis of their length, and (**B**) types of tRFs (tRF-5, tRF-3 and tRF-1) identified for each plant species.

For PtRFdb, we analyzed a total of 1344 sRNA sequencing datasets of 10 different plant species and identified three different types of tRFs. We used an in-house bioinformatics pipeline for the analysis of sequencing datasets (Figure 3). After this analysis, a total of 487 765 tRFs entries (258 439 tRF-5, 225 380 tRF-3 and 3946 tRF-1) were generated and incorporated in PtRFdb (Figure 4A). This dataset represents a total of 5607 unique tRFs sequences (758 tRF-1, 2269 tRF-3 and 2580 tRF-5) in the 10 plant species analyzed (Figure 4B). Distribution of different kinds of tRFs in all the plant species is represented in Table 1. Figure 5 is depicting the unique tRF sequences of different lengths ranging from 15–28 nt identified for each plant species. The length-wise distribution of unique tRF sequences is shown in Figure 6A. This figure clearly indicates that most of the tRFs are represented by length of 18–24 nt. As mentioned in the previous literature (18, 31), the number of tRF-5 is greater than tRF-3; and tRF-1 are fewer in number, similar distribution of the three types of tRFs is observed in almost all plant species (Figure 6B). Distribution of length among different type of tRFs (i.e. tRF-5, tRF-3 and tRF-1) in all plant species again reveals that the majority of tRFs lie in the range of 18–24 nt. With the best of our knowledge, prediction of tRFs was carried out in 10 plant species and their repository was successfully created containing 487 765 entries. PtRFdb is expected to be a high utility resource to the researchers involved in plant genomic studies and could help in the further identification, validation and characterization of plant tRFs.

**Table 1.** Distribution of different kind of tRFs in all the plant species

Plant	Total entries	Total unique tRFs	tRF-5	tRF-3	tRF-1
*Arabidopsis thaliana*	219 164	2923	1382	1278	263
*Brachypodium distachyon*	13 812	847	395	421	31
*Glycine max*	146 694	1348	595	614	139
*Medicago truncatula*	11 980	631	396	220	15
*Oryza sativa*	2554	168	111	42	15
*Physcomitrella patens*	5490	508	189	207	112
*Populus trichocarpa*	3078	195	107	88	0
*Sorghum bicolour*	5068	433	226	151	56
*Vitis vinifera*	70 599	893	476	373	44
*Zea mays*	9326	701	407	205	89

## Limitations and update of PtRFdb

Although we have analyzed the sequencing data generated by NGS technologies to identify the tRFs present in 10 plant species, experimental validation should be done for further characterization of tRFs. In future, the availability of sRNA sequencing datasets with tRNA gene information for other plants may lead to the further identification of tRFs in diverse plant species. Attempts will be made to update this database regularly twice a year.

## Supplementary data

Supplementary data are available at *Database* Online.

## Supplementary Material

Supplementary Data
